# Kindlin-2 loss in condylar chondrocytes causes spontaneous osteoarthritic lesions in the temporomandibular joint in mice

**DOI:** 10.1038/s41368-022-00185-1

**Published:** 2022-07-04

**Authors:** Yumei Lai, Wei Zheng, Minghao Qu, Christopher C. Xiao, Sheng Chen, Qing Yao, Weiyuan Gong, Chu Tao, Qinnan Yan, Peijun Zhang, Xiaohao Wu, Guozhi Xiao

**Affiliations:** 1 Department of Orthopedic Surgery, Rush University Medical Center, Chicago, IL USA; 2 Department of Orthopaedic Center, Xinjiang Production and Construction Corps Hospital, Urumqi, China; 3 Department of Biochemistry, School of Medicine, Southern University of Science and Technology, Guangdong Provincial Key Laboratory of Cell Microenvironment and Disease Research, Shenzhen Key Laboratory of Cell Microenvironment, Shenzhen, China; 4 Department of Orthopedics, Union Hospital, Tongji Medical College, Huazhong University of Science and Technology, Wuhan, China

**Keywords:** Ageing, Oral diseases, Cartilage

## Abstract

The progressive destruction of condylar cartilage is a hallmark of the temporomandibular joint (TMJ) osteoarthritis (OA); however, its mechanism is incompletely understood. Here, we show that Kindlin-2, a key focal adhesion protein, is strongly detected in cells of mandibular condylar cartilage in mice. We find that genetic ablation of Kindlin-2 in aggrecan-expressing condylar chondrocytes induces multiple spontaneous osteoarthritic lesions, including progressive cartilage loss and deformation, surface fissures, and ectopic cartilage and bone formation in TMJ. Kindlin-2 loss significantly downregulates the expression of aggrecan, Col2a1 and Proteoglycan 4 (Prg4), all anabolic extracellular matrix proteins, and promotes catabolic metabolism in TMJ cartilage by inducing expression of Runx2 and Mmp13 in condylar chondrocytes. Kindlin-2 loss decreases TMJ chondrocyte proliferation in condylar cartilages. Furthermore, Kindlin-2 loss promotes the release of cytochrome c as well as caspase 3 activation, and accelerates chondrocyte apoptosis in vitro and TMJ. Collectively, these findings reveal a crucial role of Kindlin-2 in condylar chondrocytes to maintain TMJ homeostasis.

## Introduction

The temporomandibular joint (TMJ) is commonly affected by the TMJ disorders (TMD).^1^ Recently, TMD has become one of the most prevalent musculoskeletal diseases and affects ~5%–12% of the population worldwide.^2^ Osteoarthritis (OA) is the most commonly reported and serious subtype of TMDs, especially in women and older people.^3,4^ In mainland China, it has been reported that about 14.56% of TMD patients displayed radiographic signs of OA.^5^ The main structure of TMJ consists of the articular disc, mandibular condyle, glenoid fossa, and a capsule that covers the joint. The major pathological features of TMJ OA can be characterized by progressive cartilage degradation, chondrocyte hypertrophy, inflammation, and osseous changes, such as subchondral sclerosis and growth of bone spurs.^1,4,6^ The clinical symptoms of TMJ OA include chronic orofacial pain and joint dysfunction.^1,4,6^ During the last decade, a range of genetically modified mouse models has been generated to investigate the regulatory mechanisms of TMJ OA.^7^ Alterations in expression and/or activation of Wnt/β-catenin, fibroblast growth factors (FGFs), nuclear factor kappa-light-chain-enhancer of activated B cells (NF-κB) and MTORC1 pathways, and abnormal and sustained mechanical loading have been linked to the pathogenesis of TMJ OA.^7,8^ However, the molecular mechanisms underlying the initiation and progression of TMJ OA remain incompletely understood.

Kindlin family proteins are a group of focal adhesion (FA)-related proteins, which are involved in a number of cellular processes such as cell adhesion, migration, proliferation, and signal transduction.^9–17^ To date, three Kindlin proteins have been identified in mammals, termed Kindlin-1, -2, and -3, which have distinct tissue-specific expression patterns. Kindlin-1 is mostly found in epithelial cells, including intestinal epithelial cells and keratinocytes, while Kindlin-3 is mostly found in the spleen, thymus nodes, dendritic cells, lymph, macrophages, but not in the brain, cardiac tissues, kidney, liver, testis or skeletal muscle.^18–20^ In contrast, Kindlin-2 is ubiquitously expressed excepting hematopoietic cells and is the only Kindlin protein found in embryonic stem cells.^21^ Cumulating evidence has shown that Kindlin-2 exerts multiple important functions in control of organ and tissue formation as well as homeostasis.^22–34^ For instance, we have previously reported that Kindlin-2 expression is essential for chondrogenesis and skeletal development through its mediation on TGF-β signaling pathway and Sox9 expression.^30^ However, whether Kindlin-2 is produced by adult condylar chondrocytes and whether Kindlin-2 functions in these cells for the maintenance of TMJ homeostasis remain unknown.

In the present study, we find that Kindlin-2 expression is strongly detected in condylar chondrocytes and dramatically downregulated in aged mice. Interestingly, Kindlin-2 ablation in these cells causes multiple spontaneous osteoarthritic lesions in TMJ in adult mice. Kindlin-2 loss impairs TMJ homeostasis by inhibiting chondrocyte proliferation and accelerating chondrocyte apoptosis. Furthermore, deletion of Kindlin-2 reduces anabolic ECM protein expression while increasing catabolic ECM protein expression in condylar cartilages in mice.

## Results

### Kindlin-2 is strongly detected in mandibular condylar chondrocytes in mice

**Fig. 1:**
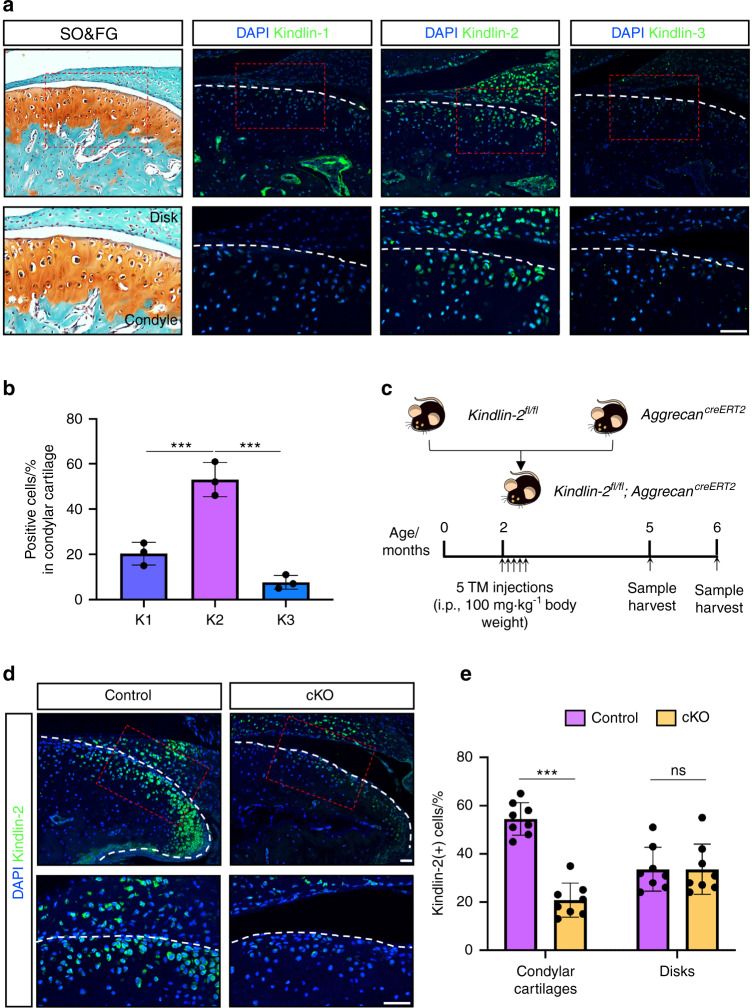
The high protein expression level of Kindlin-2 in mandibular condylar chondrocytes in mice. **a** Representative images of safranin O & fast green (SO&FG, left panel) and immunofluorescent (IF, right panels) staining of mouse TMJ sections. Higher-magnification images (red dashed boxes) are shown in lower panels. Scale bar: 50 μm. **b** Quantification of Kindlin-1-, 2-, and -3-positive cells in condylar cartilage. *N* = 3 mice per group. ***P < 0.001. **c** A schematic diagram illustrating the experimental design. **d** IF staining for Kindlin-2 expression in control or cKO TMJs at 3 months post TM injections. Scale bar: 50 μm. **e** Percentages of Kindlin-2-expressing cells in mandibular condylar cartilage and articular disc, respectively. Results are expressed as mean ± standard deviation (s.d.). *n* = 8 mice per group. ***P < 0.001; ns not significant, TM tamoxifen

We determined the expression pattern of three different Kindlin proteins in mouse TMJ. TMJ specimens were collected from 3-month-old C57BL/6 male mice and histological and immunofluorescent (IF) staining analyses were performed. Our data revealed a high protein expression level of Kindlin-2 in mandibular condylar chondrocytes in TMJ (Fig. 1a, b). Conversely, Either Kindlin-1 or Kindlin-3 was almost undetectable in condylar chondrocytes (Fig. 1a). Notably, Kindlin-1 was also detected in subchondral bone tissues.

### Genetic ablation of Kindlin-2 in condylar chondrocytes at adult stage induces multiple spontaneous OA-like phenotypes in TMJ

We next investigated whether Kindlin-2 expression is essential in condylar chondrocytes. We crossed *Kindlin-2*^*fl/fl*^ mice with the *Aggrecan*^*CreERT2*^ transgenic mice to produce *Kindlin-2*^*fl/fl*^*; Aggrecan*^*CreERT2*^ (referred to as *K2*^*fl/fl*^*; Aggrecan*^*CreERT2*^) mice (Fig. 1c). Sixteen 8-week-old male *K2*^*fl/fl*^*; Aggrecan*^*CreERT2*^ mice were intraperitoneally injected with tamoxifen (TM) (100 mg·kg^−1^ body weight per day, 5 injections) for conditional deletion of *Kindlin-2* gene in aggrecan-expressing chondrocytes (hereinafter referred to as cKO) (Fig. 1c). Of note, another 16 age-matched male *K2*^*fl/fl*^*; Aggrecan*^*CreERT2*^ mice were administrated with corn oil and served as control group. At 3 and 4 months post tamoxifen treatments, the mice were killed and TMJ specimens were collected (*n* = 8 mice/group for each time point). IF staining analyses confirmed that the protein expression of Kindlin-2 was markedly downregulated in condylar chondrocytes, but not in cells of the articular disc, in cKO TMJs relative to control TMJs (Fig. 1d, e).

**Fig. 2:**
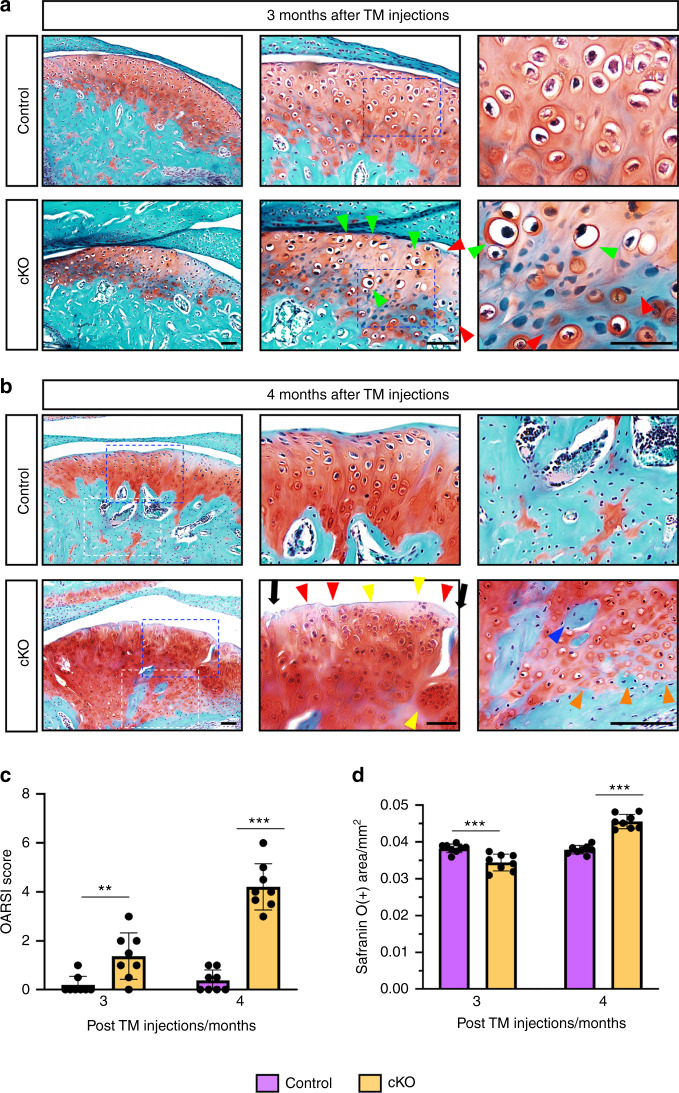
Kindlin-2 deficiency causes condylar cartilage lesions in TMJ in adult mice. **a** SO&FG staining of control or cKO TMJs at 12 weeks after TM treatments. Blue dashed boxes indicate the higher-magnification images in right panels. Scale bar: 50 μm. **b** SO&FG staining of control or cKO TMJs at 16 weeks after TM treatments. Blue dashed boxes indicate the articular cartilage area and white dashed boxes indicate the subchondral bone area. Green arrows indicate the hypertrophic articular chondrocytes. Red arrows indicate the loss of Safranin O-positive cartilage. Orange arrows indicate the ectopic cartilage formation. Blue arrows indicate the new woven bone formation within the hypertrophic chondrocyte areas. Black arrowheads indicate the fissures on condylar cartilage surface. Yellow arrows indicate the appearance of disorganized rounded chondrocytes. Scale bar: 50 μm. **c** Quantitative analyses of the Osteoarthritis Research Society International (OARSI) score. Results are expressed as mean ± standard deviation (s.d.). *N* = 8 mice per group. **P < 0.01; ***P < 0.001. **d** Quantitative analyses of Safranin O-positive areas in TMJ sections from control and cKO mice. Results are expressed as mean ± standard deviation (s.d.). *n* = 8 mice per group. TM tamoxifen

**Fig. 3:**
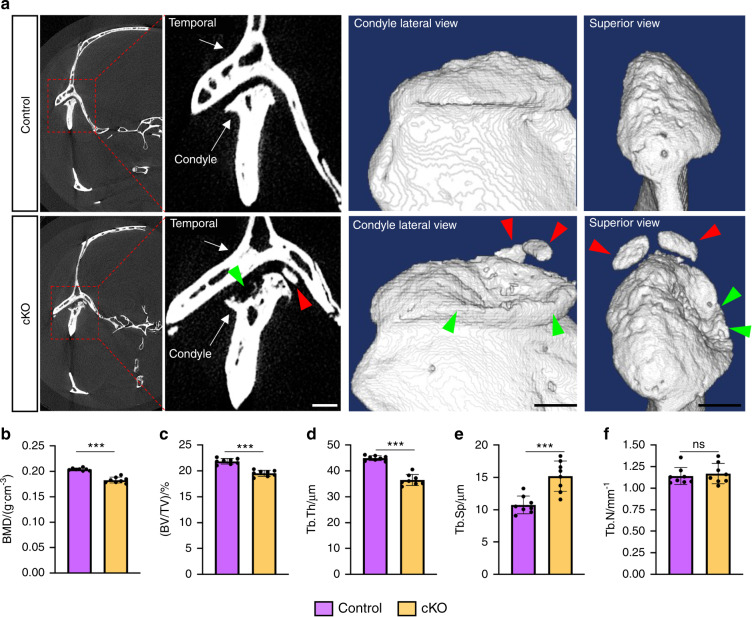
Kindlin-2 deficiency causes subchondral bone damage and ectopic bone formation in mouse TMJ. **a** Representative micro-computed tomography (μCT) sections of TMJ from control and cKO mice at 16 weeks after TM treatments (left panels) and three-dimensional (3D) reconstructions of the condyles (right panels). Scale bar, 1.5 mm. Red arrowheads indicate ectopic bone formation in cKO TMJ. Green arrowheads indicate the subchondral bone damage in cKO TMJ. **b**–**f** Quantitative μCT analysis of the bone mineral density (BMD), bone volume/tissue volume (BV/TV), trabecular thickness (Tb.Th), trabecular separation (Tb.Sp) and trabecular number (Tb.N) of the mandibular condyles. *n* = 8 mice per group. ***P < 0.001; ns not significant

We next performed safranin O & fast green staining on TMJ sections and the results showed that cKO mice displayed early signatures of OA in TMJ as early as 3 months post TM treatments, including decreased number of chondrocytes in superficial, middle, and deep layers with less safranin O staining in these areas (Fig. 2a, red arrows) and increased amounts of hypertrophic chondrocytes in superficial and middle layers (Fig. 2a, green arrows). At 4 months after TM injections, severe OA lesions, including spontaneous surface fissures at the superficial and middle layers (Fig. 2b, black arrowheads), loss of safranin O staining (Fig. 2b, red arrows), appearance of numerous disorganized rounded chondrocytes at superficial and deep layers (Fig. 2b, green arrows), and massive ectopic cartilage formation followed by new bone formation in mandibular condyle (Fig. 2b, blue arrows), were observed in cKO mice. Quantitative analyses revealed significantly higher Osteoarthritis Research Society International (OARSI) scores in cKO TMJs as compared with those in control TMJs (Fig. 2c) (*P* < 0.01, Student’s *t*-test). In addition, the safranin O-positive-stained cartilages in TMJs were decreased at 3 months, but markedly increased at 4 months, after TM injections (Fig. 2d). Subchondral bone damage and ectopic bone formation in TMJ were observed in cKO group at 4 months post TM treatments, as demonstrated by micro-computerized tomography (μCT) analyses (Fig. 3a). Moreover, quantitative μCT data showed that, when compared to control mice, the cKO mice displayed significantly decreased bone mineral density (BMD) (Fig. 3b), bone volume/tissue volume (BV/TV) (Fig. 3c) and trabecular thickness (Tb.Th) (Fig. 3d) and increased trabecular separation (Tb.Sp) (Fig. 3e) with no markedly altered trabecular number (Tb.N) (Fig. 3f). Taken together, the above findings demonstrate that genetic ablation of Kindlin-2 in aggrecan-expressing condylar chondrocytes results in severe osteoarthritic lesions in TMJ in adult mice.

### Kindlin-2 loss reduces the expression of anabolic ECM proteins in condylar cartilages

**Fig. 4:**
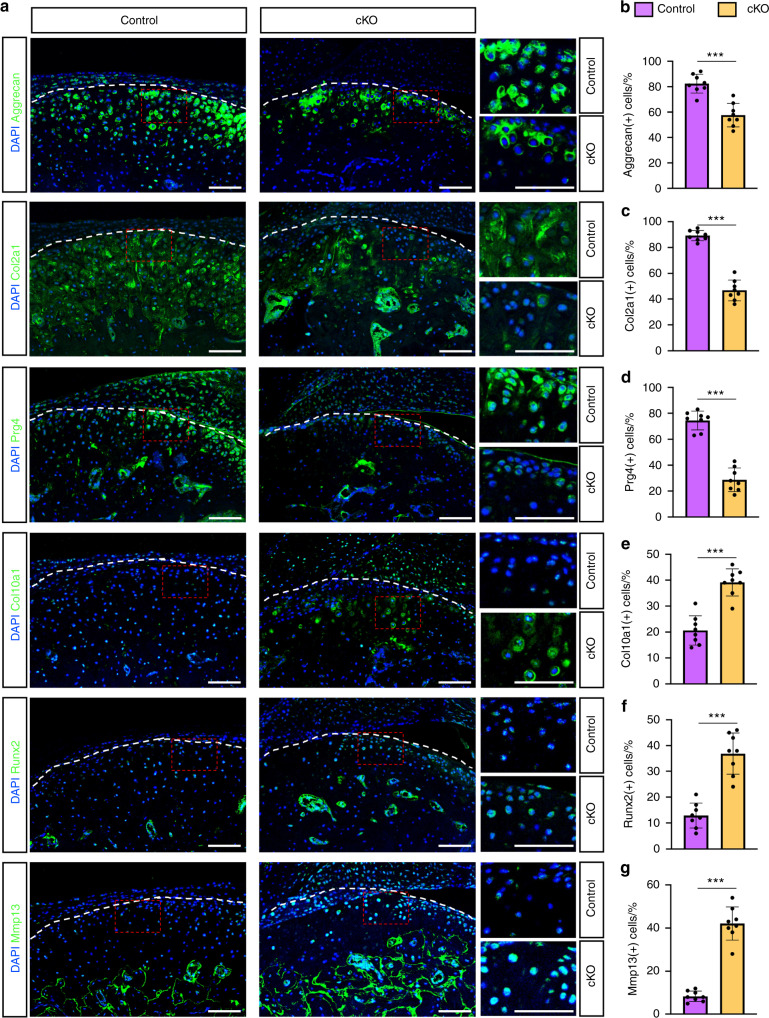
Kindlin-2 loss causes condylar chondrocyte hypertrophy and ECM degradation in condylar cartilage. **a** IF staining for expression of Aggrecan, Col2a1, Prg4, Col10a1, Runx2, and Mmp13 in control or cKO TMJs at 12 weeks post TM treatments. Red dashed boxes indicate the higher-magnification images in right panels. Scale bar: 50 μm. **b**–**g** Quantitative data of Aggrecan- (**b**), Col2a1- (**c**), Prg4- (**d**), Col10a1- (**e**), Runx2 (**f**), and Mmp13-expressing cells (**g**) in mandibular condylar cartilages of the two groups. Results are expressed as mean ± standard deviation (s.d.). *n* = 8 mice per group. ***P < 0.001

We found that the expression levels of anabolic ECM proteins, including aggrecan, Col2a1 and Prg4, were all dramatically reduced in condylar cartilages of cKO mice, as demonstrated by IF staining analyses. (Fig. 4a). Quantitative analyses showed that the percentages of aggrecan-, Col2a1- and Prg4-positive cells were decreased by 24.75%, 42.5% and 45.75%, respectively, in cKO condylar cartilages vs. control condylar cartilages (Fig. 4b–d) (*P* < 0.001, cKO vs. control for all indicated parameters, Student’ *t*-test).

### Kindlin-2 deficiency promotes condylar chondrocyte hypertrophy and ECM degradation in condylar cartilages

During the development of OA, abnormal expression of Runx2 was reported to enhance chondrocyte hypertrophic differentiation, upregulate the expression of chondrocyte hypertrophic marker Col10a1, and lead to excessive production of ECM-degrading enzymes, such as matrix metalloproteinase (Mmp13).^35,36^ We found that expression of Runx2, Col10a1 and Mmp13 was extremely low in superficial and middle layers of condylar cartilages in control mice (Fig. 4a), which were all significantly upregulated in these areas in cKO relative to control mice (Fig. 4a, e–g) (*P* < 0.001, cKO group vs. control group for all quantitative parameters, Student’ *t*-test). Collectively, these data suggest that genetic ablation of Kindlin-2 in condylar chondrocytes enhances the hypertrophic differentiation of condylar chondrocytes and ECM degradation in TMJ cartilage.

### Kindlin-2 loss inhibits condylar chondrocyte proliferation in TMJ

**Fig. 5:**
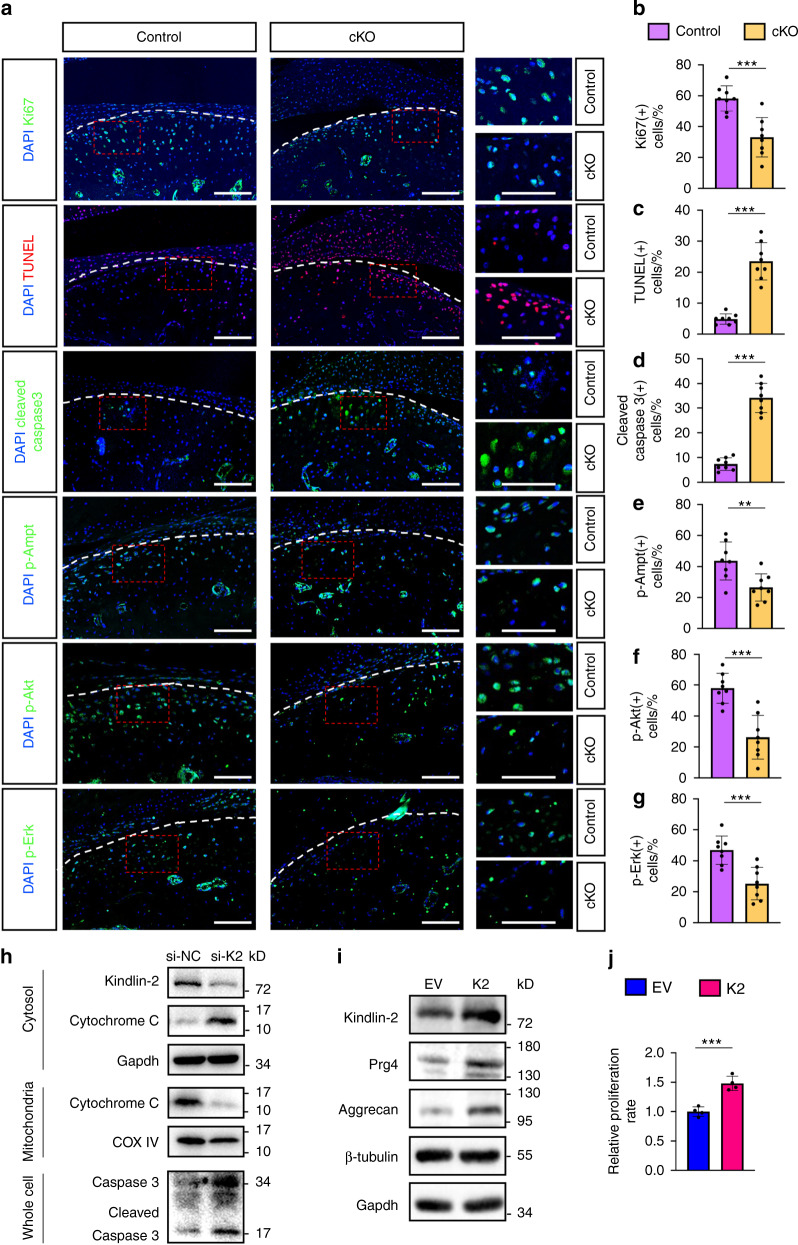
Kindlin-2 loss decreases chondrocyte proliferation and induces chondrocyte apoptosis in condylar cartilage. **a** Fluorescent staining of Ki67, TUNEL, cleaved Caspase 3, p-Ampk, p-Akt and p-Erk in control or cKO TMJ sections at 12 weeks after TM treatments. Right panels show the higher-magnification images (red dashed boxes). Scale bar: 50 μm. **b**–**g** Quantitative data of **a**. *n* = 8 mice per group. **h** Western blotting. Protein extracts isolated from cytosol, mitochondria, or whole cells of cultured ATDC5 cells after transfection of negative control siRNA (si-NC) or Kindlin-2-targeting siRNA (si-K2). **i** Western blotting analyses of protein extracts from ATDC5 cells after transfection of either Kindlin-2-expressing vector (K2) or empty vector (EV). **j** Cell proliferation rate normalized to the EV group. All in vitro experiments were independently repeated at least three times with similar results. Results are expressed as mean ± standard deviation (s.d.). **P < 0.01; ***P < 0.001

We further performed IF staining of cell proliferation marker Ki67 to assess whether the proliferation activity of condylar chondrocytes is affected by Kindlin-2 deletion. In control TMJs, Ki67 was highly expressed in condylar chondrocytes (Fig. 5a). However, the numbers of Ki67-positive chondrocytes were decreased by 25.2% in the superficial and middle layers of condylar cartilages in cKO TMJs compared to those in control TMJs (Fig. 5b) (*P* < 0.001, cKO vs. control, Student’ *t*-test).

### Kindlin-2 loss accelerates cell apoptosis in condylar chondrocytes in TMJ and in cultured ATDC5 cells

At 3 months after TM injection, Kindlin-2 loss significantly increased condylar chondrocyte apoptosis, as demonstrated by the terminal deoxynucleotidyl transferase-mediated nick-end labeling (TUNEL) staining of TMJ sections of the two groups (Fig. 5a, c). Furthermore, expression of cleaved caspase 3, a critical executioner of intrinsic apoptosis, was significantly upregulated in condylar chondrocytes of cKO vs. control mice (Fig. 5a, d) (7.37% ± 2.50% in control group vs. 34.12% ± 5.98% in cKO group, *P* < 0.001, Student’s *t*-test). In contrast, the expression levels of p-Ampk, p-Akt and p-Erk were all markedly decreased in condylar chondrocytes of cKO mice compared with those in control mice (Fig. 5a, e–g), revealing a dysregulation of signaling pathways involved in cartilage homeostasis and survival caused by Kindlin-2 loss. To explore the underlying mechanism, we knocked down Kindlin-2 expression in ATDC5 chondrogenic cells by siRNA and found that it dramatically reduced the protein level of mitochondrial cytochrome c, a critical factor that is involved in mitochondrial apoptosis,^37^ and concomitantly increased the expression of cytosolic cytochrome c (Fig. 5h). In support of an increase in cell apoptosis, Kindlin-2 knockdown by siRNA significantly increased the levels of both total and cleaved caspases 3 proteins in ATDC5 cells (Fig. 5h). These findings demonstrate that loss of Kindlin-2 expression stimulates chondrocyte apoptosis in vitro and in TMJ.

### Overexpression of Kindlin-2 enhances the production of ECM proteins and cell proliferation in ATDC5 cells

Next, we investigated whether overexpression of Kindlin-2 affects the expression of ECM proteins and cell proliferation in ATDC5 cells. Our data showed that overexpression of Kindlin-2 markedly enhanced the production of ECM proteins, such as Prg4 and Aggrecan, in ATDC5 cells (Fig. 5i). Moreover, we found that Kindlin-2 overexpression significantly enhanced the cell proliferation rate of ATDC5 cells (Fig. 5j).

### Reduced Kindlin-2 expression in TMJ chondrocytes in aged mice

**Fig. 6:**
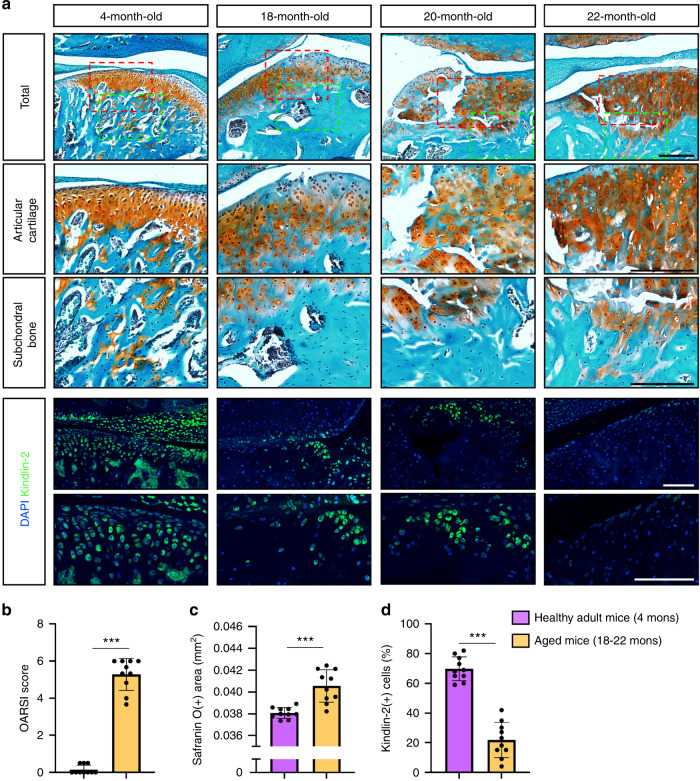
Kindlin-2 expression is downregulated in TMJs in aged mice. **a** Representative SO&FG and IF staining of TMJ sections from healthy adult (4 months) and aged (18–22 months) mice. Higher-magnification images (red and green dashed boxes) are shown in lower panels. Scale bar: 50 μm. **b** OARSI score. **c** Safranin O-positive areas. **d** Percentage of Kindlin-2-positive cells in TMJ sections from adult and aged mice. Results are expressed as mean ± standard deviation (s.d.). *n* = 10 mice per group. ***P < 0.001

Aging is the most important independent risk factor for developing OA.^38^ We next determined the expression level of Kindlin-2 in TMJs in aged mice. TMJ samples were collected from healthy adult (4 months) or aged (18–22 months) mice and subjected to histological and IF staining. As expected, aged mice display multiple OA lesions in TMJs, including severe cartilage damage, surface fissures, ectopic cartilage formation, and subchondral bone sclerosis (Fig. 6a). Furthermore, the OARSI scores and safranin O-positive areas were higher in aged TMJs as compared with those in healthy adult TMJs (Fig. 6b, c). Importantly, we found that the protein level of Kindlin-2 was significantly lower in aged TMJs than in healthy adult TMJs, as demonstrated by IF staining analyses (Fig. 6d).

## Discussion

In the present study, we demonstrate that the FA-related protein Kindlin-2, through its expression in aggrecan-expressing condylar chondrocytes, plays an important role in control of TMJ homeostasis in adult mice. Specifically, we demonstrate a high protein expression level of Kindlin-2 in TMJ condylar chondrocytes in adult mice, which is dramatically decreased in aged mice. Our data show that Kindlin-2 deficiency in aggrecan-expressing condylar chondrocytes results in a series of osteoarthritic lesions in TMJ in adult mice. This is the first demonstration of the critical role of Kindlin-2 in regulation of condylar chondrocyte function and TMJ homeostasis.

The progressive cartilage destruction and osteophyte formation are hallmarks of human TMJ OA; however, the underlying molecular mechanisms remain poorly defined.^7^ Studies from different groups by using genetic mouse models showed that abnormalities in expression and activation in factors in Wnt/β-catenin, Runx2, FGF, MTORC1 signaling pathways are involved in initiation and progression in TMJ OA. For examples, Hui and co-workers reported that activation of β-catenin in Col2a1- or aggrecan-expressing chondrocytes causes multiple osteoarthritic defects in mouse TMJ, including aberrant chondrocyte hypertrophic differentiation, condylar cartilage degradation, rough articular surface and subchondral bone sclerosis.^39,40^ Wang et al. reported that Fgfr1 deficiency in Col2a1-expressing chondrocytes protects against TMJ OA progression through enhancing autophagy of condylar chondrocytes in TMJ.^1^ We previously showed that abnormal and sustained mechanical loading accelerated TMJ OA development and progression by activation of the MTORC1 pathway.^8^ In this study, we provide a piece of new knowledge in this field by demonstrating that the Kindlin-2 signaling exerts pivotal functions in maintaining TMJ homeostasis in mice. We provide compelling evidence that genetic ablation of Kindlin-2 in condylar chondrocytes induces progressive TMJ cartilage loss and deformation, which is followed by massive ectopic new cartilage and bone formation in adult mice. Our data show that genetic ablation of Kindlin-2 in condylar chondrocytes also causes severe subchondral bone damage and ectopic bone formation in mouse TMJ. These Kindlin-2 loss-induced OA-like phenotypes in mouse TMJ highly mimic the major pathological characteristics of human TMJ OA. Thus, this Kindlin-2 loss-induced TMJ OA mouse model may serve as a novel spontaneous disease model for studying TMJ OA-related pathological mechanisms in the field of oral biology.

Our findings imply that Kindlin-2 loss causes cartilage degradation and TMJ OA through, at least in part, induction of chondrocyte apoptosis. We provide several lines of evidence to support this notion. First, loss of Kindlin-2 significantly increases condylar chondrocyte apoptosis in TMJ. Second, Kindlin-2 loss upregulates the expression level of cleaved caspase 3 both in vitro and in vivo. Third, Kindlin-2 loss decreases the expression levels of p-Ampk, p-Akt and p-Erk in condylar chondrocytes. Fourth and most importantly, we find that Kindlin-2 loss induces cytochrome c release from mitochondrion to cytosol, which is known to be a critical step for the initiation of cell apoptosis. Interestingly, our previous studies have demonstrated that Kindlin-2 is primarily localized in the mitochondria of human A549 non-small cell lung cancer cells.^28^ Moreover, genetic deletion of Kindlin-2 in these cells increases reactive oxygen species (ROS) production and apoptosis.^28^ While these findings clearly demonstrate that Kindlin-2 loss induces mitochondrial apoptosis, the underlying mechanisms require further investigations.

It should be noted that alterations in the articular disc were observed in cKO TMJs at 3 months post TM treatments, as revealed by IF staining. Hui and co-workers have reported a high Cre-recombination efficiency of *Aggrecan*^*CreERT2*^ in condylar chondrocytes, but not in cells of articular disc, after TM injections.^39^ Consistently, our data show that TM-induced *Aggrecan*^*CreERT2*^ activation significantly reduced the expression of Kindlin-2 in condylar chondrocytes, but not in cells of articular disc.^41^ It is well known that OA is a whole joint disease. Kindlin-2 loss-induced cartilage deformation might indirectly affect the articular disc, for instance, by changing the mechanical distribution and/or micro-environment of TMJ. The underlying mechanisms need to be elucidated in greater detail in future studies.

Although TMJ disorders mostly affect young women, it has been reported that the prevalence and severity of TMJ OA increase dramatically in older population.^42^ In fact, aging is one of the most important independent risk factors for developing OA.^38^ In this study, we find that the protein expression of Kindlin-2 is drastically downregulated in TMJ cartilage of aged mice as compared with that in adult mice. The TMJ osteoarthritic damages induced by Kindlin-2 deletion are highly similar to the aging-induced OA damages in mice, suggesting a potential involvement of reduced expression of Kindlin-2 in condylar chondrocytes during the development and progression of aging-related TMJ OA.

It should be noted that the condylar cartilage area as measured by safranin O- staining analysis was decreased at 3 months, but markedly increased at 4 months, after TM injections in cKO mice. The observed differences in cartilage content were attributed to the loss of cartilage in superficial and middle layers at the early stages of Kindlin-2 loss-induced TMJ OA, which was followed by ectopic cartilage formation in subchondral areas. Interestingly, we also detected massive ectopic cartilage formation in TMJs of aged mice. These findings suggest that ectopic cartilage formation may play a key role in the pathogenesis of TMJ OA, which warrants further investigations.

There are limitations in this study. First, we did not determine the expression level of Kindlin-2 in human TMJ. Whether Kindlin-2 expression is downregulated in human TMJ OA samples needs to be determined. At the present, it is difficult to obtain human TMJ samples for this study. Second, since TMJ is a load-bearing joint, it will be interesting to determine if and how Kindlin-2 deficiency impacts the OA lesions in abnormal mechanical loading models, such as unilateral anterior crossbite model.^43^ Third, while our results clearly show that Kindlin-2 loss induces multiple striking osteoarthritic lesions in TMJ, whether overexpression of Kindlin-2 in mouse TMJ can exert protective effects against TMJ OA development and progression remains to be determined.

Based on our findings of this study that Kindlin-2 expression is dramatically reduced in condylar chondrocytes of aged TMJ and that Kindlin-2 loss causes osteoarthritic lesions in mouse TMJ that highly mimic those in human TMJ OA, along with the fact that abnormal and sustained mechanical stress plays a critical role in pathogenesis of TMJ and knee joint OAs and established role of the focal adhesion pathway in mediation of mechanotransduction in skeleton,^8,29,44–46^ it will be crucial to investigate if changes in Kindlin-2 expression, especially in condylar chondrocytes, play a role in the pathogenesis of TMJ OA in humans.

In conclusion, our study reveals that Kindlin-2 expression in aggrecan-expressing condylar chondrocytes is essential for maintaining TMJ homeostasis in mice.

## Materials and methods

### Animal model

Floxed *Kindlin-2* (*Kindlin-2*^*fl/fl*^) mice were bred with the *Aggrecan*^*CreERT2*^ knock-in mice to obtain the *Kindlin-2*^*fl/fl*^*; Aggrecan*^*CreERT2*^ mice. For conditional knockout of *Kindlin-2* gene in aggrecan-expressing chondrocytes, sixteen 2-month-old male *Kindlin-2*^*fl/fl*^*; Aggrecan*^*CreERT2*^ mice were intraperitoneally administrated with tamoxifen (Sigma T5648, 100 mg/kg body weight per day, 5 injections). Another 16 age-matched male *Kindlin-2*^*fl/fl*^*; Aggrecan*^*CreERT2*^ mice administrated with corn oil were used as controls. This study was approved by the Institutional Animal Care and Use Committees (IACUC) of the Southern University of Science and Technology.

### Quantitative histopathological analyses

TMJ specimens were harvested from mice immediately after sacrifice and kept in 4% paraformaldehyde solution overnight at 4 °C. The decalcification, dehydration, and paraffin embedding of TMJ samples were performed following our previously established protocols.^8,32^ The paraffin-embedded TMJ samples were cut into 5-micron sections and stained using a safranin O & fast green staining kit (Solarbio, Cat#G1371) as previously described.^23^ The SO&FG-stained sections were evaluated according to the OARSI scoring system in a double-blinded manner. The Safranin O-positive cartilage areas were determined by image J (version 1.53k) as previously described.^47^

### Immunofluorescent analyses

For immunofluorescent (IF) staining, 5-micron TMJ sections were hydrated and permeabilized for 5 min at room temperature (RT). The TMJ sections were blocked with for 1 h at RT and then probed with primary antibodies in a humidifying box overnight at 4 °C. The slices were washed in phosphate-buffered saline with 0.1 percent Tween 20 and then stained with fluorescence-labeled secondary antibodies (Invitrogen, Cat# A-11008) for 1 h at RT. The fluorescence signals on TMJ sections were analyzed by Leica SP8 Confocal Microsystems.

### Quantitative μCT analyses

Quantitative μCT analyses were performed using a Skyscan scanner 1276 μCT system (Bruker, Belgium) as previously described.^24,44,48^

### TUNEL staining

Cell apoptosis was assessed using a commercial TUNEL assay kit (Beyotime, C1090) according to the manufacturer’s guidelines.^49,50^

### In vitro siRNA knockdown experiments

ATDC5 cells were cultured in DMEM/F12 supplemented with 5% FBS, 1% penicillin and streptomycin, and 1% insulin-transferrin-selenium (Gibco™, Cat# 51500056) to induce chondrogenic differentiation. For in vitro knockdown of Kindlin-2 expression, ATDC5 cells were transfected with Kindlin-2-targeting siRNA as previously described (Invitrogen, Cat# 13778075) as previously described.^51^ ATDC5 cells transfected with a negative control siRNA were used as control group. Protein extracts were collected 48 h after siRNA transfection and examined by western blotting. The Kindlin-2-targeting siRNA sequences: 5’ primer-GUGGCUAGAUUCCUCAAGATT, 3’ primer-UCUUGAGGAAUCUAGCCACTT.

### Extraction of cytosolic and mitochondrial fractions from cultured ATDC5 cells

We used a commercial mitochondria isolation kit (Thermo Fisher Scientific, Cat #898874) to extract the cytosolic and mitochondrial fractions from cultured ATDC5 cells, as previously described.^28^

### Statistical analysis

Mice were randomly assigned to each group in this study. The Prism GraphPad software was used to perform statistical analysis. The quantitative data were presented as mean ± standard deviation (s.d.). For statistical analysis, the two-tailed unpaired Student’s *t*-test was used. *P* < 0.05 were considered statistically significant.

## Data Availability

All data generated for this study are available from the corresponding authors upon reasonable request.
